# Associations of Single Nucleotide Polymorphism in *miR-146a *Gene with Susceptibility to Breast Cancer in the Iranian Female

**DOI:** 10.31557/APJCP.2020.21.6.1585

**Published:** 2020-06

**Authors:** Elham Siasi, Mahkameh Solimani

**Affiliations:** *Department of Genetic, Collage of Sciences, North Tehran Branch, Islamic Azad University, Tehran, Iran. *

**Keywords:** Breast cancer, Single nucleotid polymorphism (SNP), miR146a gene- rs2910164, Tetra ARMS PCR

## Abstract

**Background:**

MicroRNAs (miRNAs), short regulatory RNAs, function as negative regulators able to modulate gene expression. Just as other genetic variant, single nucleotide polymorphisms (SNPs) in *miRNA* genes, may have an impact on their expression and/or maturation and hence leading to different consequences in carcinogenesis. Accordingly, this study aimed to assess the frequency of *miR-146a G/C* (*rs2910164*) polymorphism and its association with susceptibility to breast cancer in Iranian women.

**Methods:**

We conducted a case-control study using Tetra ARMS polymerase chain reaction (Tetra ARMS PCR) method in 100 Iranian female participants (50 breast cancer patients and 50 controls). Besides, a number of sequenced samples were chosen to confirm the accuracy of genotyping by Tetra ARMA PCR. SPSS software was utilized for all statistical analyses. The odds ratios (ORs) and 95% confidence intervals (95% CIs) were applied to analyze the association between the SNP frequency and breast cancer.

**Results:**

The frequency of genotypes for G/G, G/C, and C/C were 23 (46%), 26 (52%), and 1 (2%) among cases and 15 (30%), 33 (66%), and 2(4%) among controls, respectively. The results generated by the groups did not show any significant correlation between *miR-146a G/C *(*rs2910164*) polymorphism and breast cancer, either at genotype or allele levels (P>0.05). F-SNP-based in silico analysis indicated possible modifications in transcriptional regulations induced by miR-146a G/C (rs2910164) variations.

**Conclusion:**

Overall, our results indicated no correlation between miR-146a G/C (rs2910164) polymorphism and genetic susceptibility to breast cancer in Iranian female populations. However, these findings need to be further confirmed by analyses of a larger number of cases.

## Introduction

Breast cancer is the most prevalent cancer among women worldwide and the leading cause of cancer-related death in less-developed countries (Bansal et al., 2014). Along with the environmental factors and lifestyle that potentially increase the risk of breast cancer, there are genetic factors implicated in the pathogenesis of the disease. The recent discovery of genetic alterations involved in the disease has attracted undivided attention in breast cancer research. Thus far, many of such variants have been identified with potential associations to the risk of breast cancer (Lian et al., 2012). By way of illustration, the mutations in the* BRCA1/2* genes are responsible for an average risk of 16–20% for hereditary breast cancer. Recent research, however, has produced evidences to support the role microRNAs (miRNAs) play in development and progression of breast cancer (Morales et al., 2018). The human genome encodes more than 1,000 miRNAs (Wang et al., 2013). They are noncoding, short (usually 21–23 nucleotides in length), and evolutionarily conserved, RNA molecules that exert post-transcriptional regulation as they bind to complementary sequences in 3′-untranslated region (3′ UTR) or 5′-untranslated region (5′UTR) of target messenger RNAs (mRNAs) (Mu et al., 2017). It is estimated that around 30% of human genes are regulated by miRNAs (Wang et al., 2013). The genetic variants of a miRNA could affect its biogenesis including its maturation, and is likely to have a role in the pathogenesis of many diseases, such as cancer (Wang et al., 2013). As almost 50% of genes encoding miRNA happen to be in cancer-related chromosomal regions, the latest studies have investigated their potential use as cancer susceptibility and prognosis markers (Mu et al., 2017). For instance, the result of a study (Lian et al., 2012) showed that there were 39 interactions between *BRCA1 *mRNA and microRNA-146a, introducing this miRNA as a negative regulator of *BRCA1*. Among different variations in human genome, single-nucleotide polymorphisms (SNPs) are the most prevalent (Morales et al., 2018). In the case of miRNAs, such SNPs might change their expression, lead to maturation of aberrant miRNA, and affect the target binding affinity and specificity of transcription (Mu et al., 2017). Therefore, they can be potentially linked to human malignancies. As breast cancer is the most reported one among females, it has become a major area of concern for studies linking miRNAs and human cancers (Dai et al., 2016). In this regard, numerous epidemiological studies evaluated the association between cancer susceptibility and miRNA SNPs (Jazdzewski et al., 2008; Xu et al., 2008; Jazdzewski et al., 2009; Guo et al., 2010; Xu et al., 2010; Zeng et al., 2010; Permuth-Wey et al., 2011). Furthermore, several meta-analyses case-control studies carried out on the association focused on breast cancer in different populations (Jedlinski et al., 2011; Linhares et al., 2012; Zhang et al., 2012; Omrani et al., 2014; Qi et al., 2015; Morales et al., 2016). For example, the human *miR-146a* gene at locus 5q34 was linked with BRCA1/BRCA2 activity. The SNP rs2910164:G>C, located in the middle of the miRNA stem hairpin, was reported to cause an alteration in the expression of this miRNA, since it replaces a G:U pair with a C:U mismatch in the stem of the miRNA precursor (Lian et al., 2012; Mu et al., 2017; Mu et al., 2017; Morales et al., 2018). This SNP was shown to increase the risk of some human cancers including carcinoma of the breast, urinary bladder, cervical, head and neck, gastric, and lung (Jazdzewski et al., 2008; Xu et al., 2008; Jazdzewski et al., 2009; Guo et al., 2010; Xu et al., 2010; Zeng et al., 2010; Permuth-Wey et al., 2011). Specifically, several molecular studies were conducted on the link between the miR-146a rs2910164 polymorphism and the increased/decreased risk of breast cancer (Catucci et al., 2010; Pastrello et al., 2010; Garcia et al., 2011; Lian et al., 2012; Wang et al., 2013; Bansal et al., 2014; Chen et al., 2014; He et al., 2015; Upadhyaya et al., 2016; Afsharzadeh et al., 2017; Mu et al., 2017; Afzal et al., 2018; Morales et al., 2018). Here, we analyzed the association between miR-146a rs2910164 G>C polymorphism and breast cancer susceptibility in Iranian females population by means of Tetra ARMS PCR. 

## Material and Methods

*Participants*

In this case-control study, we recruited 50 patients with histologically confirmed breast cancer and 50 healthy controls. The controls were frequency-matched to patient cases in terms of age, gender, as well as ethnicity. We used medical records and cancer registry data to confirm self-reported cases of breast cancer, and to gain information on tumor characteristics. The cases and the controls were unrelated, yet of similar ethnicity in Iran. The study was approved by Islamic Azad University Ethics Committee (Cod No. 15730503961004) and the authors followed the norms of the World’s Association Declaration of Helsinki. The study methods were carried out according to approved guidelines. Informed consent was obtained from all the participants. The sample size of the case and control groups was calculated with standardized Cochrane formula (as below): 

$n=\frac{\frac{z^{2}\mathrm{pq}}{d^{2}}}{1+\frac{1}{N}[\frac{z^{2}\mathrm{pq}}{d^{2}}-1]}$

n=sample size

N=population size

P=proportion of population

q=1-p

z=1.96

d=margin of error (0.05)

*DNA Extraction and Genotyping*

To meet the sampling requirements, about 3 mL of venous blood was collected from each participant. The blood samples were collected in tubes containing ethylene diamine tetra acetic acid (EDTA). Subsequently, the samples were centrifuged at 8,000g for 180s at room temperature. After centrifugation, the plasmas were stored at 80°C. Genomic DNA from each sample was isolated from the leucocytes of the peripheral blood using a DNA Purification Kit (CinnaGen, Iran) according to the manufacturer’s instructions. Genotyping of miR-146a rs2910164 G/C polymorphism was performed through Tetra ARMS PCR. As presented in Table 1 the primers used for this study were the same as the ones applied by Afzal (2018), synthesized by Sinaclon, Iran. PCR reaction materials (Fermentas, Iran) and thermal cycles stages (with BioRad-T100 Termal cycler) are presented in Tables 2 and 3. 

*Sequencing*

In order to confirm the PCR genotyping results, some samples were randomly chosen and sequenced by Bioneer Corporation (Korea).

*Statistical and Bioinformatics Analysis*

Statistical analysis was performed by SPSS version 24. Descriptive statistics of patients and controls were presented as the mean and standard deviations for continuous measures, and frequencies and percentages for categorical measures. A chi-square test was run to calculate the additive combined effects of the risk alleles G and C. The odd ratios (Ors) with their corresponding 95% CIs (homozygote comparison, heterozygote comparison, dominant model and recessive model, respectively) were calculated to analyze the associations of polymorphism rs2910164 in miR-146 with breast cancer susceptibility and testing the Hardy-Weinberg Equilibrium (HWE). The cut-off p-value for the association was <0.05. Bioinformatics analysis was performed by bioinformatics tools FAST-SNP (http://fastsnp.ibms.sinica.edu.tw) and F-SNP (http://compbio.cs.queensu.ca/F-SNP/).

## Results

*Characteristic Profile of the Participants*

**Figure 1:**
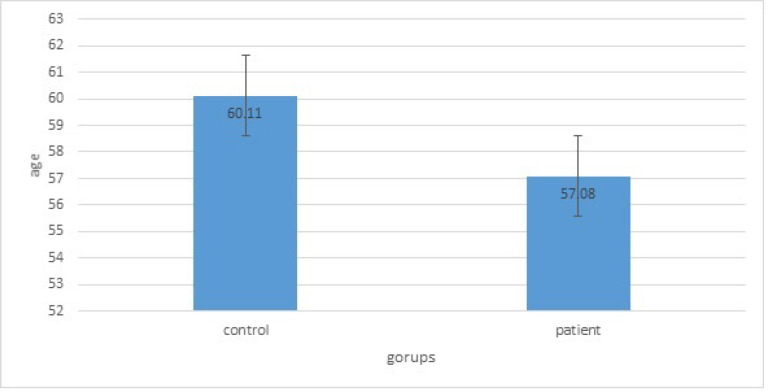
Average Age for Control and Patient Groups

**Figure 2:**
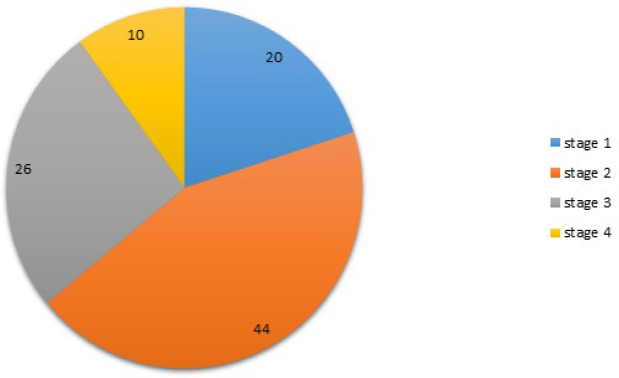
Stage Frequency of Breast Cancer Degree in 50 Patients

The average age for case (50 breast cancer patients) and control (50 controls) groups were 49.09±11.02 and 48.80±8.28 years, respectively (Figure 1). We found that the genotyped related to rs2910164 variant was significantly more frequent in older subjects. Figure 2 shows stage frequency of breast cancer degree in the patient group.

*Tetra ARMS PCR Genotyping Results*

**Figure 3:**
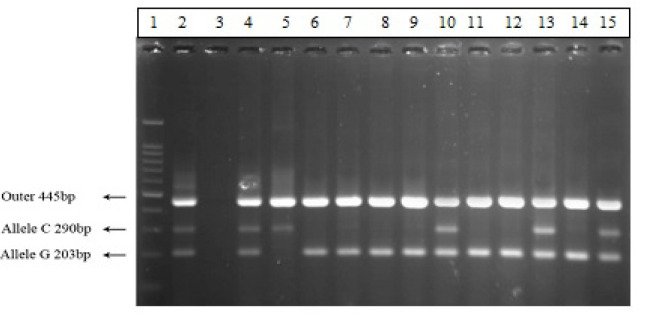
Tetra ARMS PCR Products Result of rs2910164 (G>C) in miR-146a Gene on 2% Electrophoresis Gel. Number 1: Molecular marker (100 bp), number 2: Positive control, number 3: Negative control, numbers 4,10,13,15 : GC heterozygote (203 bp, 290 bp, 445 bp), numbers 5: CC homozygote (mutant type)(290 bp, 445 bp) and numbers 6,7,8,9,11,12,14: GG homozygote (wild type)(203 bp, 445 bp).

All products of Tetra ARMS PCR were analyzed using agarose gel electrophoresis (1.5%), stained by ethidium bromide and visualized on UV transilluminatore (Figure 3).

*Between Group Comparison of the Association of miR-146a G/C (rs2910164) Polymorphism*

Table 4 represents he genotypes and alleles frequencies of the miR-146a rs2910164 G/C polymorphism in patients and controls. An *χ*^2^ test indicated the lack of any significant difference between genotype frequencies of the two settings (patients vs. control) (P>0.05). A logistic regression test was used in order to examine odds ratio for different genotypes. In addition, different patterns of inheritance (dominant, recessive, codominant, over-dominant and log additive) were analyzed as well. Dominant model was the only pattern for which the odds ratio was significant (CI95%) (Table 5). Patient and control frequency of genotypes were also tested for Hardy-Weinberg equilibrium. The result of HWE test of showed that both groups were within Hardy-Weinberg equilibrium (P<0.05) (Table 6).

*Co-relation of microRNA Polymorphism miR-146a (rs2910164) G>C with Clinical Characteristics (tumor stage) in Breast Cancer Patients*

When segregated in accordance with the tumor stage, breast cancer patients did not show any significant association of all selected variants of miR-146a (rs2910164) G>C (P>0.05).

*Sequencing Result*

**Figure 4:**
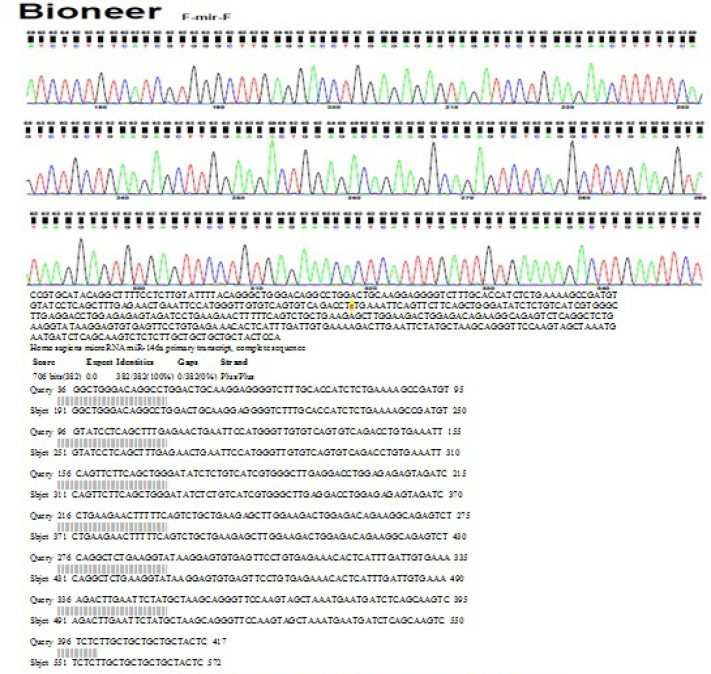
Sequencing Result for Mutant Genotype of *miR-146a* Gene rs2910164 SNP

Sequencing results were analyzed by the sequencer v5.4.6 and then were blasted using National Center for Biotechnology Information (NCBI) BLAST tool. Genotyping results by sequencing were identical to the Tetra ARMS PCR results. Sequencing result for mutant genotype of* miR-146a* gene rs2910164 SNP are shown in Figure 4.

*Bioinformatic Analysis*

The Silico analysis by Golden Plath prediction Tool (http://genome.ucsc.edu/) (F-SNP and FAST-SNP) indicated the possible effect of miR-146a G/C (rs2910164) variation on transcriptional regulation (Functional score 0.101) (Table 7).

## Discussion

MiRNAs, a class of endogenous, are non-coding, single-stranded RNAs involved in many biological processes, such as development, apoptosis, differentiation, proliferation, immune response and regulation of gene expression in post translation levels (Morales et al., 2018). Many miRNAs have been implicated in various human diseases, and it has been suggested that miRNAs are aberrantly expressed or mutated in many types of cancers (Jazdzewski et al., 2008; Xu et al., 2008; Jazdzewski et al., 2009; Guo et al., 2010; Xu et al., 2010; Zeng et al., 2010; Permuth-Wey et al., 2011). The most frequent variations in human genome; i.e. polymorphisms in miRNA, can be altred to different miRNA biology pathways including expression, processing, and maturation, as well as attraction and specificity for target binding (Ryan et al., 2010; Chen et al., 2014). Therefore, many epidemiological studies have focused on the correlation between miRNA polymorphisms and susceptibility to the most common tumors in women, i.e. breast cancer (Catucci et al., 2010; Pastrello et al., 2010; Garcia et al., 2011; Lian et al., 2012; Wang et al., 2013; Bansal et al., 2014; Chen et al., 2014; He et al., 2015; Upadhyaya et al., 2016; Afsharzadeh et al., 2017; Mu et al., 2017; Afzal et al., 2018; Morales et al., 2018). Accordingly, there has been an association documented between rs2910164 polymorphism in miRNA146a and the increased risk of several types of human cancers including papillary thyroid cancer, hepatocellular cancer, esophageal, squamous cell cancer, gastric cancer, prostate cancer, and glioma (Jazdzewski et al., 2008; Xu et al., 2008; Jazdzewski et al., 2009; Guo et al., 2010; Xu et al., 2010; Zeng et al., 2010; Permuth-Wey et al., 2011). As demonstrated by some studies, this SNP could be related with breast cancer susceptibility (Catucci et al., 2010; Pastrello et al., 2010; Garcia et al., 2011; Lian et al., 2012; Wang et al., 2013; Bansal et al., 2014; Chen et al., 2014; He et al., 2015; Upadhyaya et al., 2016; Afsharzadeh et al., 2017; Mu et al., 2017; Afzal et al., 2018; Morales et al., 2018). This SNP is present on the 3′ UTR strand of the miRNA and triggers a G to C change, leading to an alteration from a G: U pair to a C: U mismatch, which is located on the stem structure of the miRNA precursor. This encourages an alteration in miR-146a expression and thus could influence the risk of cancer (Jazdzewski et al., 2009; Lian et al., 2012; Wang et al., 2013;Mu et al., 2017; Morales et al., 2018). 

In this study, we investigated miR-146a rs2910164 SNP in 50 cases and 50 controls. In our study, we found that the Iranian population data is different with other populations. As with other research projects, the results of our study found ethnicity as a source of discrepancy between the data on the Iranian and other populations (Jazdzewski et al., 2009; Catucci et al., 2010; Pastrello et al., 2010; Garcia et al., 2011; He et al., 2015; Afsharzadeh et al., 2017). We did not find any relation between this polymorphism and risk of breast cancer in Iranian women. Our findings were in agreement with those of some other studies (Shen et al., 2008; Catucci et al., 2010, GAO et al., 2011; Wang et al., 2012). 

However, a study by Lian et al., (2012) explained that CC genotype of this SNP was able to increase the risk of breast cancer in European populations. Shen et al., (2008) reported the first case of correlation between this SNP and breast cancer, in terms of a correlation between rs2910164 and the patients with familial breast/ovarian cancer. In addition, Pastrello et al., (2010) reported rs2910164 to have a potential impact on the breast cancer in an Italian population. Bansal et al., (2014) found that the rs2910164 G/G polymorphism was associated with reduced genetic susceptibility to breast cancer in a population from North India. There was also a group of postmenopausal breast cancer patients reported without any familial history of the disease, where some genotypes of rs2910164 in miR-146a were correlated to an increased risk of breast cancer in a Chinese population (He et al., 2015). 

Between 2011 and 2017, several meta-analyses trading were published with rs2910164 G>C polymorphism were published (Jazdzewski et al., 2008; Srivastava et al., 2012; Wang et al., 2013; Chen et al., 2014; Mu et al., 2017; Zhang et al., 2017). Among them, two studies reported rs2910164 SNP is related to breast cancer susceptibility in a Caucasian population (Jazdzewski et al., 2008; Srivastava et al., 2012). The other five studies showed that rs2910164 was not correlated to breast cancer susceptibility in both Caucasian and Asian populations (Gao et al., 2011; Wang et al., 2013, Chen et al., 2014; Mu et al., 2017; Zhang et al., 2017). In another meta-analysis, Lian et al., (2012) initiated a significant correlation between the rs2910164 CC genotype and the risk of breast cancer in European populations. In contrast, a research by Hu et al., (2009) did not indicate an association between this SNP and the risk of breast cancer in Chinese women. More recently, two studies were dedicated to the relationship of this polymorphism and the breast cancer among different ethnicities of the Iranian population (Nejati-Azar et al., 2017; Mashayekhi et al., 2018). The former analyzed the association of miRNAs 146aG/C (rs2910164) polymorphism with the risk of breast cancer between case and control groups of Azeri ethnicity women in Iran. Their analysis, similar to our research, showed no significant relationship between miRNAs 146aG/C (rs2910164) polymorphism and breast cancer susceptibility in Azeri ethnicity in Iran (Nejati-Azar et al., 2017). The latter, however, indicated that the CC homozygous genotype of miR-146a (rs2910164) was seen in 45 (12.7%) patients with breast cancer and could be related to breast cancer in Rasht ethnicity in Iran (Mashayekhi et al., 2018). 

What mentioned above simply indicates that the genetic background could have a deep impact upon oncogenic mechanisms across populations. Therefore, further studies in larger populations including different ethnicities, and other genetic and environmental factors, such as genetic markers, assortment and socioeconomic sections, are required to achieve a definitive conclusion concerning the probable relationship of this polymorphism with breast cancer in any populations including the Iranian one. 

Nevertheless, what may merit attention to review the limitations and strengths of our study. The very first point is that while genetic associated studies concerning common diseases normally enjoy large datasets, small p values and independent replication for more reliable results the study involved a very small number of cases and controls. When sufficiently large (n>=30), the sample statistically benefits from a normally distributed sample mean that allows a z test to be run for a single mean. Yet, a small sample size (n<30) calls for running a t-test to compensate for the absence of a normal distribution.

The second area of concern with association studies is if the design allows the researchers to replicate the tests. This ultimately ensures the accuracy of the results and enhances the degree of significance. In order for the results to be reliable, it is essential to pay meticulous attention to factors like confounding population structure, misclassification of outcome, allelic heterogeneity and gene-disease associations that might in theory limit the application of this approach. 

Another serious problem with association studies is that population structure can lead to false associations between a candidate marker and a phenotype. Although for a study of associations between genotype and the risk of a disease, the case-controls are created to the cohort the design, cohort studies have been recommended because they can be used to study gene-environment interactions. Despite the pertinent scientific relevance of its statistical interaction, there is a very considerable disadvantage in using the case-control design susceptibility to bias when estimating effects of exposures that are measured retrospectively. The pitfall is that such a process does not necessarily apply to the cases where the investigation targets the statistical interaction between genotype and environmental exposure. According to the latest designs run for genetic association studies, it is highly recommended to rely on randomized sampling in order to compare the relation between genotypes (heterozygote and homozygotes) and environmental risk factors. To rely on random selection or to choose a cohort design are other measures to increase the reliability of the results in genetic experiments with a small sample size. The reason is that such measures could substantially control both the impact of environmental factors on the disease related genes and the sensitivity of other disruptive factors. These, in return, minimize the risk of error and significantly compare the interactions between genotypes and environmental risk factors. In brief, randomized sampling (of both the cases and controls) will assist to achieve adequate statistical power (or a significant statistical analysis) required to detect the factors that might have an impact on the above mentioned relation, particularly when the risk factors are unknown. The randomized sampling is as valuable as the cohort design. Subsequently, this study enjoyed a randomized sampling method and a cohort design. 

Next comes the discussion concerning the significance of the p value for Hardy Weinberg equilibrium test. The results of our study demonstrated that both control and case groups were within Hardy-Weinberg equilibrium (P<0.05); this also could be explained by the small sample size. In this regard, it has been suggested that the gene associated with human disease show enormous variation (in the number and population frequency of the disease predisposing alleles at the loci) in their allelic spectra. For some genes, there are a few predominant disease alleles, whereas others have a wide range of disease alleles, each relatively rare. The allelic spectrum is important, disease genes with only a few deleterious alleles can be more readily identified and are more amenable to clinical testing. It has become increasingly apparent that the identification of true genetic associations in common multifactorial disease calls for studies comprising thousands rather than the hundreds of individuals employed so thus. 

When the sample size is small, it can be difficult to see if there is any difference between the sample mean and the population mean, because the amounting degrees of sampling variability act as a source of confusion. Thus, it is highly recommended to resolve this matter before the standard error could be calculated, and the standard deviation could be divided by the sample size. Besides, when the sample size is small, it is desirable to consider the probabilities of both type I and type II errors as this will allow to use a larger alpha for the purpose of justification. With a small sample, the probability of a type II error with the standard alpha of 0.05 may be too high, and might act in a way appropriate to an occasion when in the face of a p value greater than 0.05, the null hypothesis is false. However, when the p value is greater than 0.05, there is less evidence for the false null hypothesis than the time there is a smaller p value with a larger sample. Here, the rationale is that a p value greater than 0.05 with a small sample is just strong evidence against the null hypothesis as a smaller p value with a big sample. Moreover, when there is only a minimum sample size, regardless of total number of population, one can consider using t statistic with the appropriate degrees of freedom as reliable means of performing advanced statistical tests. This approach is efficient even at presence of a tiny sample size (especially for n<20). In addition, restricting the statistical test results to 95% confidence interval enables researchers to generalize their results to the complete population with a similar range to that of the total population. Furthermore, mathematical likelihood (odds ratio, CI95%), shows that the p value substantially overstates the evidence against the null hypothesis. The likelihood depicts the distinction between error rates and inferential evidence more clearly and as a quantitative tool for expressing the evidential strength, it outperforms the p value for the purposes of epidemiological purposes.

Finally, as breast cancer is a heterogeneous disease, we had to thoroughly investigate any potential gene-gene interaction and gene-environment interactions. As indicated by a large number of researchers in the area of breast cancer, variation in the frequency of allelic variants of human genes, can affect not only the risk itself, but the assessment of the associated risk scales as well. Similarly, some studies focused on genotype prevalence and gene disease associations, where these focuses of research was on sample selection, analytic validity of genotyping, population stratification, and statistical issues. The growing body of research on genetic markers as well as the development of more sophisticated tools for genotyping have recently made a considerable contribution to proliferation of association studies. Therefore, the time has come to critically reconsider the design of such studies, in order to avoid the mistakes of the past and maximize their potential to identify new components of diseases.

 Metabolic and signaling pathways that regulate hormone action are two potential aspects of the breast cancer, demanding undivided attention. When perturbed, they simply alter their outputs of matter and energy which, depending on the environmental context, can produce either a pathological or a normal phenotype. To investigate the dynamics of these pathways through approaches like metabolic control analysis may provide new insights into the pathogenesis and treatment of complex diseases. One of this control mechanisms, is microRNAs. Variations and changing expressions of microRNAs (miRNAs), could bring about multiple changes in the expression of tumorigenesis promoting genes that ultimately increase the risk of breast cancers. Recent data on breast cancer-specific miRNA polymorphisms and expression profiles and their participation in regulating invasive processes, illustrated that they were associated with changes in cytoskeletal structure, cell-cell adhesion junctions, cancer cell-extracellular matrix interactions, tumor microenvironments, epithelial-to-mesenchymal transitions and cancer cell stem abilities. Besides, breast cancer invasiveness are shown to be strongly triggered by epigenetic regulation of individual miRNAs and their modified interactions with other regulatory genes, and with the functions of miRNA isoforms and exosome-mediated miRNA. Research into miRNA’s function in breast cancer has just begun and there is still a long way to go. 

This has roots in two important issues: First, most genotypes that contribute to the constellation of necessary genes are uncommon and hard to recognize. Second, although more common genotypes may confer susceptibility, they are weak predictors of particular diseases such as breast cancer. Consequently, more attention must be inevitably paid to environmental risk factors. For this reason, more important than conducting a genetic search, is acquiring the know ledge about the relations among environmental / genetic factors and other interactions. For example, obesity–insulin connections have been considered as potential risk factors for postmenopausal breast cancer, and the association between insulin resistances (IR) genotypes and phenotypes can be modified by obesity-lifestyle factors, affecting breast cancer risk. Recently, more and more studies, have confirmed accurate predictions of breast cancer and indicated potential intervention strategies for women with specific genetic and lifestyle factors (exercise, diet, smoking, safe habit, alcoholism and sufficient relaxation) to correlate them with breast cancer risk. In this study, the results indicated that there was relation between older age and breast cancer risk in Iranian female. Finally, multiple comparisons are commonly made in genetic association research, yet how to appropriately change for multiple comparisons remains a controversial issue. However, that large increases in the number of comparisons has a limited effect on the sample size required to maintain an experiment level.

In summary, our results suggested that the rs2910164 (G > C) polymorphism in miR-146a may not be associated with increased susceptibility to breast cancer and could not be a biomarker to predict the breast cancer risk and potential therapeutic targets in the studied population. However, the mechanisms essential for this association need to be further discovered. Further studies need to focus on the role of genetic variants of miRNA in breast cancer and inclusion of diverse ethnic groups and larger sample sizes in order to confirm the role of rs2910164 polymorphism in miR-146a with breast cancer pathobiology.

**Table 1:** Primers for Amplification of *miR-146a* Gene rs 2910164 SNP

Primer	Sequence 5´ - 3´	PCR product
Primer-F (Allele-C)	5´- tccatgggttgtgtcagtgtcagagctc-3´	290bp
Common Primer-R	5´- gagtagcagcagcagcaagagagactt-3´	
Common Primer-F	5´- tagacctggtactaggaagcagctgcat-3´	203bp
Primer-R (Allele-G)	5´- atatcccagctgaagaactgaattacac-3´	
Common Primer-F	5´- tagacctggtactaggaagcagctgcat-3´	445 bp
Common Primer-R	5´- gagtagcagcagcagcaagagagactt-3´	

**Table 2:** Reagents and Volumes of PCR Reaction Mix (in 25 μl)

Reagent	Volume (μl)
Master mix 2X	10
Forward Primers	1 (× 2)
Reverse Primers	1 (×2)
DNA	4
H_2_O	7
Totals	25

**Table 3:** Termocycle Program for Amplification of *miR-146a *Gene rs2910164 SNP

Reaction Step	Tm (°C)	Time	Cycle
Pre denaturation	95	5 min	1
Denaturation	95	30 sec	
Annealing	59	30 sec	35
Extension	72	90 sec	
Final extension	72	10 min	1

**Table 4:** Genotypes Frequency of miR-146a rs2910164 G/C Polymorphism in Case and Control Gorups

Genotype	Frequency in controls	Frequency in cases	Total	Allele	Frequency in controls	Frequency in cases	Total	*P*-value
GG	15 (30%)	23 (46%)	38	G	32 (64%)	36 (72%)	68	
GC	33 (66%)	26 (52%)	59					0.391
CC	2 (4%)	1 (2%)	3	C	18 (36%)	14 (28%)	32	
Total	50 (100%)	50 (100%)	100		50 (100%)	50 (100%)	100	

**Table 5:** Inheritance Models miR-146a rs2910164 G/C Polymorphism and Odds Ratio (OR) with 95% Confidential Interval between Case and Control Groups

Model	Genotype	Controls	Cases	*P*-value
Dominant	GG	15 (30%)	23 (46%)	0.098
	GC+CC	35 (90%)	27 (96%)	
Recessive	CC	2 (4%)	1 (2%)	0.554
	GG+GC	48 (96%)	49 (98%)	
Overdominant	GC	33 (66%)	26 (52%)	0.154
	GG+CC	17 (34%)	24 (48%)	
	GG	15 (30%)	23 (46%)	
Codominant	GC	33 (66%)	26 (52%)	0.391
	CC	2 (4%)	1 (2%)	

**Table 6:** Test of Hardy-Weinberg Equilibrium for Control and Case Groups

Frequency	GG genotype	GC genotype	CC genotype	Totals	G allele	C allele	Totals	*P*-value
Frequency in controls	15	33	2	50	32	18	50	0.001
Frequency in cases	23	26	1	50	36	14	50	0
Totals	38	59	3	100	68	32	100	0

**Table 7.** Insilico Analysis for *miR-146a* G/C rs2910164 Polymorphism

Variation	rs2910164	Position in protein	-
Location	chr5:160485411	Amino acid change	-
Allele	G/C	Codon change	-
Gene	MIR146A	Co-located Variation	rs2910164
Feature	ENST00000385201.1	Extra	Exon=1/1
Feature type	Transcript	Functional categories	Transcriptional regulation
Consequence	Non-coding-transcript-exon-variant	Prediction Tool	GoldenPath
Position in cDNA	60	Prediction Result	Exist
Position in CDS	-	FS score	0.101
